# Nutrition in the spotlight in cachexia, sarcopenia and muscle: avoiding the wildfire

**DOI:** 10.1002/jcsm.12673

**Published:** 2020-12-31

**Authors:** Carla M. Prado, Stefan D. Anker, Andrew J.S. Coats, Alessandro Laviano, Stephan von Haehling

**Affiliations:** ^1^ Human Nutrition Research Unit, Department of Agricultural, Food and Nutritional Science University of Alberta Edmonton AB Canada; ^2^ BIH Centre for Regenerative Therapies Charité Uinversitätsmedizin Berlin Berlin Germany; ^3^ Department of Cardiology (Campus Virchow‐Klinikum) Charité Universitätsmedizin Berlin Berlin Germany; ^4^ German Centre for Cardiovascular Research (DZHK), partner site Berlin Berlin Germany; ^5^ Monash University Melbourne Australia; ^6^ University of Warwick (UK) Warwick UK; ^7^ Department of Translational and Precision Medicine Sapienza University Rome Italy; ^8^ Department of Cardiology and Pneumology University Medicine Goettingen Goettingen Germany; ^9^ German Centre for Cardiovascular Research (DZHK), partner site Göttingen Göttingen Germany

**Keywords:** Nutrition, Sarcopenia, Cachexia, Muscle, Muscle loss, Nutrition intervention, Supplements

The pathophysiology of muscle loss alone or in the context of malnutrition, sarcopenia, or cachexia is multifactorial: hormonal, neurological, inflammatory, functional/mobility, age‐related, disease‐specific, treatment‐related, and others.^1^, ^2^ Nutrition is a key factor because both quality and quantity of nutrients are essential to support muscle anabolism, lessen catabolism, and improve prognosis.^3^, ^4^, ^5^, ^6^, ^7^, ^8^, ^9^, ^10^ This is true even in the context of cachexia. Nutrition alone cannot reverse cachexia but can prevent or minimize further loss, alleviate symptoms, and improve quality of life and outcomes in general.^11^, ^12^

It is surprising that we know little about the specific nutrient needs of people with cachexia, sarcopenia, or other diseases of muscle loss. Nutrition‐related guidelines in several such diseases are based mostly on expert consensus, rarely on clinical trial evidence. There is a fundamental need to understand the optimal macronutrient and micronutrient ‘mix’ that is advised for or offered to people with these conditions.

Likewise, we know little about the synergistic or additive roles of ‘muscle‐building nutrients’ (*Figure* 1) to sustain muscle mass in muscle loss diseases. The same is true in the more neglected scenario of paediatric nutrition, where low muscle mass is emerging as an important problem with little past or ongoing research to inform clinical practice.^13^, ^14^ This lack of targeted nutrient recommendations may also impact the optimal use of nutrition strategies within multimodal interventions, which are recognised as ideal for multifactorial conditions.

**Figure 1:**
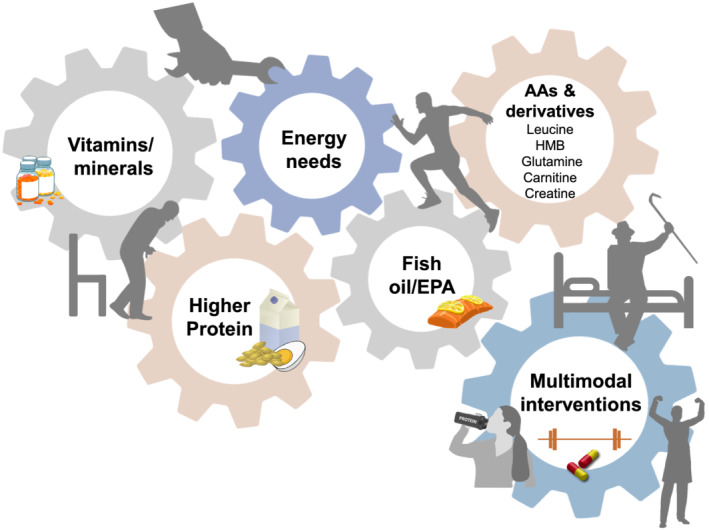
Selected nutritional approaches under consideration for treating muscle loss. AA, amino acids; HMB, β‐hydroxy‐β‐methylbutyrate; EPA, eicosapentaenoic acid. Adapted from Prado et al.^6^ Concepts to be adapted to the clinical needs of patients.

Nutrition research related to muscle loss, sarcopenia, and cachexia has been chronically underfunded, leaving many gaps and opportunities (*Figure* 2). We urge funding agencies and industry to support research to bridge and fill these gaps. We also urge researchers to include measures of nutritional status as an essential variable to be accounted for and optimised in their studies.^1^ For example, pharmacological trials should assess, control, and ideally optimize nutritional status to maximize each participant's anabolic potential. The same is true for exercise intervention studies, where nutritional requirements will likely be impacted by changes in body weight and composition. Ultimately, anabolic treatments and interventions may fail if nutrition remains inadequate.^6^

**Figure 2:**
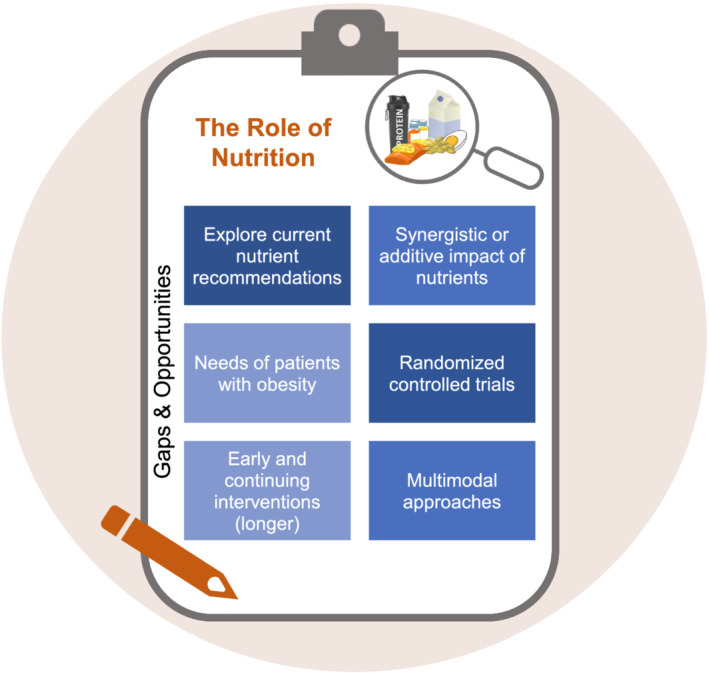
Checklist of selected gaps and opportunities around the role of nutrition in catabolic conditions.

## Avoiding the wildfire

**Figure 3:**
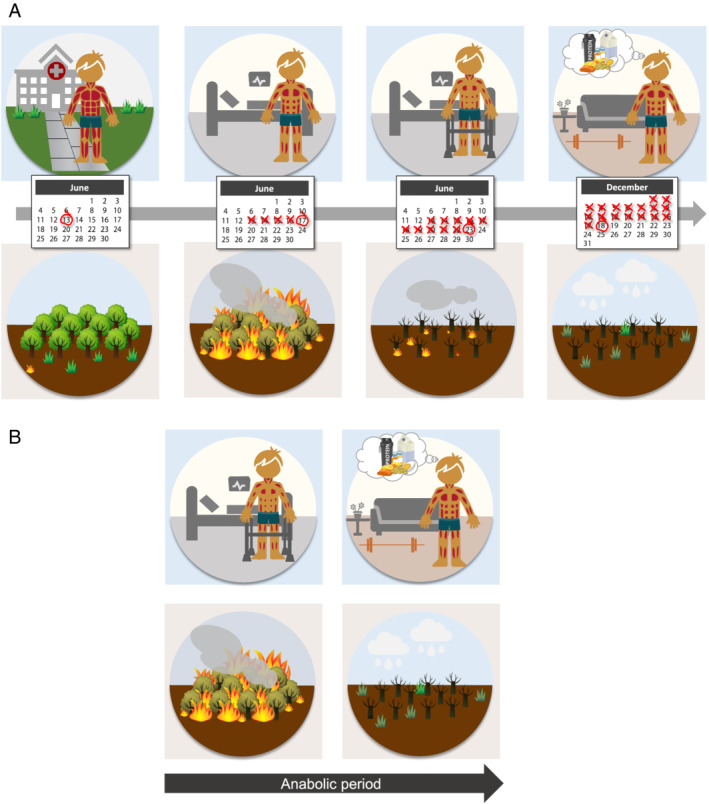
Graphic illustration of the need for early and continuing nutrition interventions for prevention and treatment of muscle loss *to be used in knowledge translation and patient education materials*. Muscle loss is a defining feature of sarcopenia and cachexia. (A) Muscle is lost rapidly, like a wildfire. Rebuilding muscle takes much longer than losing muscle, like reforestation. Summarised in (B): Long‐term interventions are needed to support the anabolic period (post wildfire). *Months in calendar are random*.

A key to nutrition intervention is *early* and *continuing* intervention. Muscle loss is a defining feature of sarcopenia and cachexia, and muscle is lost rapidly in chronic and acute conditions, especially in cachexia.^15^, ^16^, ^17^, ^18^ Conversely, muscle takes much longer to rebuild.^19^ The situation is similar to a wildfire followed by reforestation (*Figure* 3). Early intervention is essential, because preserving is better than rebuilding. From the nutritional perspective, interventions can use food, oral nutritional supplements, —enteral or parenteral nutrition as appropriate. Nutrition can also be maximised in multimodal interventions. Importantly, continuing intervention must address the changing metabolic needs of each person.

Patient education is also fundamental. An important barrier to behavioural change is that patients often do not recognize nutrition as a therapy.^20^, ^21^, ^22^ Animated videos, educational materials such as infographics, and other patient‐oriented resources (e.g. *Figure* 3) can be instrumental in educating patients about nutrition‐based therapies.^23^, ^24^ Selected examples can be watched online (at https://www.youtube.com/watch?v=pDSX_jaDCDM and https://www.youtube.com/watch?v=CAC2g03_‐2Y.

## Taking a stand: *Journal of Cachexia, Sarcopenia and Muscle* nutrition publications

We conducted a manual search of published *Journal of Cachexia, Sarcopenia and Muscle* (*JCSM*) issues from 2018, 2019, and 2020 (including ‘early view’ up to 12 December 2020) to identify human or animal studies on nutrition in sarcopenia or cachexia. We selected articles investigating nutrition interventions, macronutrient intake below recommended, and micronutrient deficiency. We found 26 articles: 10 clinical trials,^3^, ^25^, ^26^, ^27^, ^28^, ^29^, ^30^, ^31^, ^32^, ^33^ five cross‐sectional studies,^34^, ^35^, ^36^, ^37^, ^38^ three experimental animal studies^39^, ^40^, ^41^ (one of which also included a human cross‐sectional analysis^38^), three narrative reviews,^6^, ^42^, ^43^ two retrospective studies,^44^, ^45^ two systematic reviews or meta‐analyses,^46^, ^47^ and one questionnaire survey.^48^ Within the 320 original and review articles published in 2018, 2019, and 2020 in *JCSM*, the 26 articles on nutrition that we found comprise approximately 8%.

Of the nutrition studies that we found, one explored the role of protein,^31^ three explored the role of vitamin D,^26^, ^38^, ^39^ two explored the role of several nutrients,^3^, ^41^ and one explored the role of natural product (astaxanthin) supplements.^40^ Seven studies^25^, ^27^, ^28^, ^29^, ^30^, ^32^, ^33^ investigated the effects of multimodal interventions (defined as two or more approaches) on muscle mass. Articles also explored the associations of protein intake,^46^, ^47^ iron deficiency,^37^, ^42^ micronutrients,^35^ calorie restriction,^43^ nitrate dietary intake,^36^ retrospective evaluation of early dietary supplementation,^44^ and overall dietary intake and patterns^34^ with several clinical outcomes and/or biomarkers of sarcopenia or cachexia. Two studies evaluated the perceptions of oncology patients regarding disease‐related nutritional issues and barriers to effective nutritional interventions.^45^, ^48^ One narrative review discussed potential nutrition interventions to augment muscle mass.^6^

## Call for papers

Acknowledging the role of nutrition to counter cachexia, sarcopenia, and other muscle loss diseases, and the small number of publications in the topic, *JSCM* is launching a *call for papers on the role of nutrition in preventing and treating cachexia, sarcopenia, or other muscle loss diseases*. We welcome high‐quality papers of all types, but particularly original articles that explore the role of nutrition in preventing and treating these conditions.

## Conflict of interest

C.M.P. reports receiving honoraria and/or paid consultancy from Abbott Nutrition, Nutricia, Nestle Health Science, Fresenius Kabi, Pfizer, and Helsinn.

S.D.A. reports grants from Vifor Int and Abbott and personal fees from Vifor, Bayer, Boehringer Ingelheim, Novartis, Servier, Abbott, Actimed, Cardiac Dimensions, and Impulse Dynamics, all outside the submitted work.

A.J.C. has received personal fees from Astra Zeneca, Bayer, Boehringer Ingelheim, Menarini, Novartis, Nutricia, Servier, Vifor, Abbott, Actimed, Arena, Cardiac Dimensions, Corvia, CVRx, Enopace, ESN Cleer, Faraday, WL Gore, Impulse Dynamics, and Respicardia, all outside the submitted work.

A.L. reports receiving consulting fees for honoraria for lectures at industry‐sponsored events; consulting fees from Abbott, Baxter, BBraun, Fresenius Kabi, NestléHealth Science, Nutricia, and Smartfish; and research grant from Fresenius Kabi.

S.v.H. has been a paid consultant for and/or received honoraria payments from AstraZeneca, Bayer, Boehringer Ingelheim, BRAHMS, Chugai, Grünenthal, Helsinn, Hexal, Novartis, Respicardia, Roche, Sorin, and Vifor. S.v.H. reports research support from Amgen, AstraZeneca, Boehringer Ingelheim, IMI, and the German Center for Cardiovascular Research (DZHK).
